# Association of Blood NK Cell Phenotype with the Severity of Liver Fibrosis in Patients with Chronic Viral Hepatitis C with Genotype 1 or 3

**DOI:** 10.3390/diagnostics14050472

**Published:** 2024-02-21

**Authors:** Vladislav Vladimirovich Tsukanov, Andrei Anatolyevich Savchenko, Mikhail Aleksandrovich Cherepnin, Eduard Vilyamovich Kasparov, Elena Petrovna Tikhonova, Alexander Viktorovich Vasyutin, Julia Leongardovna Tonkikh, Anna Alexandrovna Anisimova, Vasily Dmitrievich Belenyuk, Alexandr Gennadyevich Borisov

**Affiliations:** 1 Federal Research Center “Krasnoyarsk Science Center” of the Siberian Branch of the Russian Academy of Sciences, Scientific Research Institute of Medical Problems of the North, 660022 Krasnoyarsk, Russia; aasavchenko@yandex.ru (A.A.S.); mikhail.cherepnin@yandex.ru (M.A.C.); clinic@impn.ru (E.V.K.); alexander_vasyutin@mail.ru (A.V.V.); tjulia@bk.ru (J.L.T.); dyh.88@mail.ru (V.D.B.); 2410454@mail.ru (A.G.B.); 2 Krasnoyarsk State Medical University Named after Prof. V.F. Voino-Yasenetsky of the Ministry of Healthcare of Russian Federation, 660022 Krasnoyarsk, Russia; tihonovaep@mail.ru (E.P.T.); tada1@mail.ru (A.A.A.)

**Keywords:** viral hepatitis C, liver fibrosis, NK cells, subsets, phenotype, receptor expression

## Abstract

Background: NK cells phenotype and functional state in different genotypes of chronic viral hepatitis C (CVHC), depending on liver fibrosis severity, have not been sufficiently studied, which limits the possibilities for the development of pathology therapy. Methods: The CVHC diagnosis was based on the EASL recommendations (2018). Clinical examination with liver elastometry was performed in 297 patients with genotype 1 and in 231 patients with genotype 3 CVHC. The blood NK cells phenotype was determined by flow cytometry in 74 individuals with genotype 1 and in 69 individuals with genotype 3 CVHC. Results: The frequency of METAVIR liver fibrosis stages F3–F4 was 32.5% in individuals with genotype 3, and 20.5% in individuals with genotype 1 CVHC (*p* = 0.003). In patients with both genotype 1 and genotype 3 CVHC, a decrease in the total number of blood NK cells, CD56^bright^CD16^+^ NK cells and an increase in the proportion of CD56^dim^CD16^+^ NK cells, CD94^+^ and CD38 ^+^ CD73^+^ NK cells were registered in patients with fibrosis stage F3–F4 by METAVIR in comparison with persons with METAVIR fibrosis stage F0–F1. Conclusions: In patients with both genotype 1 and genotype 3 CVHC, an imbalance in the ratio between cytokine-producing and cytotoxic NK cells and an increase in the content of NK cells that express inhibitory molecules were determined in patients with severe liver fibrosis.

## 1. Introduction

Currently, there are 71 million people in the world with diagnosed chronic viral hepatitis C (CVHC); 400,000 people die from this pathology every year [1]. It has been shown that the incidence of hepatocellular carcinoma (HCC) increases 15–20 times in patients with CVHC [2]. Despite advances in treatment, the problem of CVHC remains very relevant due to the remaining significant pool of patients [3,4]. The most common CVHC genotypes in Russia are the first (56.6%) and the third (35.4%) [5]. The question being debated is: in which genotype (1 or 3) does CHCV have a more aggressive course [6]? Recently, significant attention has been paid to genotype 3 CVHC in terms of understanding the pathogenetic mechanisms of HCC development and increasing the effectiveness of treatment [7,8,9].

It is traditionally considered that the antiviral immune response in hepatitis C is caused by adaptive immune system cells−T lymphocytes [10]. But with the identification in 1975 of natural killer cells (NK cells), a cellular component of innate immunity, their ability to rapidly act and powerfully respond to virus-infected cells and cancer cells was demonstrated [11,12,13]. NK cells make up 10% of the total peripheral blood lymphocyte population and represent the third largest lymphocyte population after T and B cells. [14]. A number of works have been devoted to studying the role of NK cells in CVHC [10,15,16]. It has been shown that the hepatitis C virus (HCV) affects the number and functional activity of NK cells, and therapy with protease inhibitors restores the normal adaptive phenotype and increases their production of interferon-γ [17]. The antifibrotic effect of NK cells has been established by elimination of activated stellate cells [18,19]. However, the causes of NK cell dysfunction leading to the development of liver fibrosis remain unclear [20]. The phenotype and subset composition of NK cells in different genotypes of the hepatitis C virus have not been sufficiently studied. In this regard, the study of the subset composition and functional activity of blood NK cells, depending on the severity of CVHC clinical and morphological manifestations with different genotypes of the virus, is certainly relevant.

Thus, the purpose of this research was to study association of phenotype and subset composition of NK cells with the severity of liver fibrosis in patients with chronic viral hepatitis C with genotype 1 or 3.

## 2. Materials and Methods

### 2.1. Patients

The study was conducted in the therapeutic department of the Scientific Research Institute of Medical Problems of the North (Federal Research Center “Krasnoyarsk Science Center” of the Siberian Branch of the RAS). In total, 528 patients with CVHC were examined: 297 persons with genotype 1 (164 men, 133 women, average age 41.4 years) and 231 people with genotype 3 (125 men, 106 women, average age 41.1 years). Inclusion criteria were objectively diagnosed CVHC with genotype 1 or 3 in patients aged 18 to 60 years who had not previously received treatment for CVHC and signed an informed consent for examination. The study included patients before the start of antiviral therapy. Exclusion criteria from the study were: (1) age under 18 years and over 60 years; (2) HIV infection; (3) oncological diseases; (4) genotypes 2, 4, 5, 6, 7 CVHC; (5) other chronic liver diseases of different etiologies (other viral hepatitis, opisthorchiasis, alcoholic liver disease, non-alcoholic fatty liver disease, Wilson–Konovalov disease, hemochromatosis, autoimmune hepatitis, etc.); (6) tuberculosis; (7) pregnancy; (8) serious chronic illnesses of various organs and systems; (9) narcotic drugs use; (10) patients who refused to take part in the study. All 528 patients met inclusion criteria and had no exclusion criteria.

The control group included 20 practically healthy individuals (10 men, 10 women, average age 43.3 years), in whom, during a preventive examination, serious chronic illnesses of various organs and systems were excluded, who had no complaints about their health status and denied information about alcohol abuse history, had normal complete blood count and biochemical blood test, and absence of viral hepatitis B and C markers in the blood.

According to the Helsinki Declaration on the conduct of scientific research, all subjects were informed about the objectives, methods and possible complications during the research and signed informed consents to participate in the surveys. The study was carried out with the permission of the Federal Research Center Krasnoyarsk Scientific Center SB RAS Ethics Committee (protocol No. 4 of 02.08.2019).

### 2.2. Diagnosis of CVHC

The diagnosis of CVHC was made based on results of epidemiological, clinical and laboratory examinations, including detection of specific serological markers of CVHC and HCV RNA in concordance with the European Association for the Study of the Liver (EASL) recommendations [21,22]. Determination of the virus RNA content was performed by quantitative real time polymerase chain reaction using a Biorad CFX96 Real Time System (Bio-Rad Laboratories, Hercules, CA, USA) and the Abbott RealTime HCV test^®^ system (Abbott, Abbott Park, IL, USA). The HCV genotype was determined by the VERSANT^®^ HCV Amplification 2.0 kit (LiPA, Siemens, Munich, Germany).

To diagnose associated changes and complications, all patients underwent complete blood count, biochemical blood test and ultrasound examination of the liver and pancreas. Biochemical blood tests included the determination of transaminases (ALT, AST), gamma-glutamyl transpeptidase (GGTP), alkaline phosphatase (ALP), total and direct bilirubin, total protein, albumin, iron, copper, and, if necessary, determination of ceruloplasmin. The level of CVHC activity was determined by the content of transaminases in the blood based on the Los Angeles classification of hepatitis [23]. If the presence of autoimmune hepatitis was suspected, the concentration of IgG and specific autoantibodies in the blood was measured (ASMA; LKM-1; anti-LC1).

### 2.3. Assessing the Degree of Fibrosis

The liver fibrosis degree was assessed by elastometry using the Aixplorer (SuperSonic Imagine, Aix-en-Provence, France) ultrasound system, which uses shear wave imaging. The elasticity modulus (liver stiffness) was calculated using the formula E = 3∙ρ∙Vs2, where E is the elasticity modulus in kilopascals (kPa), ρ is the density in kg/m^3^ and vs. is the velocity of shear wave propagation in m/s. Liver stiffness or vs. values gradually increase with the progression of liver fibrosis and are considered effective indicators for making the diagnosis of liver fibrosis and liver cirrhosis, in particular. Liver fibrosis was assessed using the METAVIR scale [24]. There were four degrees of fibrosis depending on the detected indicators of liver elasticity: F0—without fibrosis (≤5.8 kPa); F—(5.9–7.2 kPa), which corresponds to portal and periportal fibrosis without septa; F2—(7.3–9.5 kPa)—portal and periportal fibrosis with single septa; F3—(9.6–12.5 kPa)—portal and periportal fibrosis with multiple septa (bridge-like)—with porto-portal and portocentral septa; F4—cirrhosis (≥12.6 kPa).

### 2.4. Assessment of NK Cell Phenotype by Flow Cytometry

**Figure 1:**
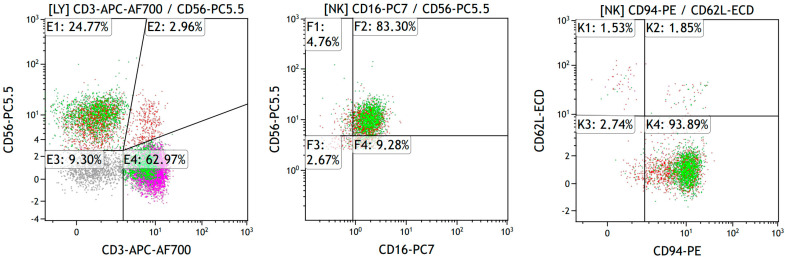
Example of tactics for gating NK cells according to panel 1. The color visualizes the distribution of cells expressing the following receptors: CD3—pink, CD16—green, CD56—red. Cells in the E3 gate that do not express CD3, CD16, and CD56 markers are shown in gray.

Venous blood samples were collected in vacuum tubes with K_3_EDTA (Becton Dickinson, Franklin Lakes, NJ, USA). The investigation of NK cell phenotype was performed no later than two hours after blood sampling. Preparation of blood samples and flow cytometer setup were carried out in accordance with the antibody manufacturers’ recommendations. Two panels of monoclonal antibodies (MATs) conjugated to different fluorochromes (all MATs manufactured by Beckman Coulter, Indianapolis, IN, USA) were used to study NK cell phenotype (Table 1). An example of the tactics for gating subsets of NK cells is shown in Figure 1. Additionally, the expression levels of functional antigens were assessed by analyzing mean fluorescence intensity (MFI). These MAb cocktails were used to stain 100 µL of blood sample in accordance with manufacturer recommendation. Removal of erythrocytes from the samples was carried out by no-wash technology with VersaLyse Lysing Solution (Beckman Coulter, Indianapolis, IN, USA, cat. A09777), 25 µL of IOTest 3 Fixative Solution (Beckman Coulter, Inc., Indianapolis, IN, USA, cat. A07800) was added to 975 µL of sample. For data analysis, Navios™ flow cytometer (Beckman Coulter, Indianapolis, IN, USA) was used, located at Krasnoyarsk Regional Center of Research Equipment of Federal Research Center Krasnoyarsk Science Center SB RAS. In each sample, at least 50,000 lymphocytes were analyzed. Cytofluorometric data were processed using Navios Software v.1.2 and Kaluza™ v.2.2 (Beckman Coulter, Indianapolis, IN, USA).

### 2.5. Funding

The study was performed within the framework of the state assignment of the Federal Research Center “Krasnoyarsk Science Center” of the Siberian Branch of the RAS, state registration number FWES-2024-0035.

### 2.6. Statistical Analysis

Quantitative parameters were presented as median (Me) and interquartile ranges (IQR), between the 25th and 75th percentiles. Clinical parameters and qualitative variables were presented as absolute values and percentages (n (%)). The significance of differences for quantitative data was assessed using the nonparametric Mann–Whitney U test. Comparison of qualitative variables was carried out using the Fisher’s exact test. Statistical analysis was performed with application the Statistica 8.0 software package (StatSoft, Tulsa, OK, USA, 2007). Graphic representation of the research results was performed using the GraphPad Prism 8.0.1 build 244 program (GraphPad Software Inc., San Diego, CA, USA, 2018). Statistically significant *p*-value was considered ≤0.05.

## 3. Results

### 3.1. Anamnestic, Clinical and Laboratory Parameters of the General Group of Patients with CVHC and Those Who Underwent Immunological Examination

We have analyzed age, gender, anthropometric data, anamnestic characteristics and laboratory parameters of the examined patients. The groups of patients were identical in age and gender. Body mass index (BMI), alcohol consumption, tobacco smoking, complete blood count data, bilirubin and albumin levels in the blood serum did not differ significantly in CVHC patients with genotypes 1 and 3. At the same time, ALT values were significantly higher in CVHC patients with genotype 3 in comparison with HCV genotype 1 individuals (Table 2). When describing groups of individuals who underwent determination of the phenotype and subset composition of NK cells using flow cytometry, no significant differences were found in age, gender, BMI, tobacco smoking, complete blood count data and serum albumin concentration in CVHC patients with genotype 1 of HCV, CVHC patients genotype 3 of HCV and control individuals. The level of ALT and bilirubin was significantly increased in patients with CVHC compared with healthy individuals. But these indicators did not differ significantly between persons with HCV genotypes 1 and 3 (Table 3).

The frequency of liver fibrosis stages F2 and F3–F4 was higher in CVHC patients with HCV genotype 3 in comparison with HCV genotype 1 persons, which in itself is an interesting fact and suggests the presence of some clinical and laboratory differences when comparing individuals with genotypes 1 and 3 of HCV (Table 4).

### 3.2. Subset Composition and Phenotype of NK Cells at Different Liver Fibrosis Degrees in Patients with CVHC

**Figure 2:**
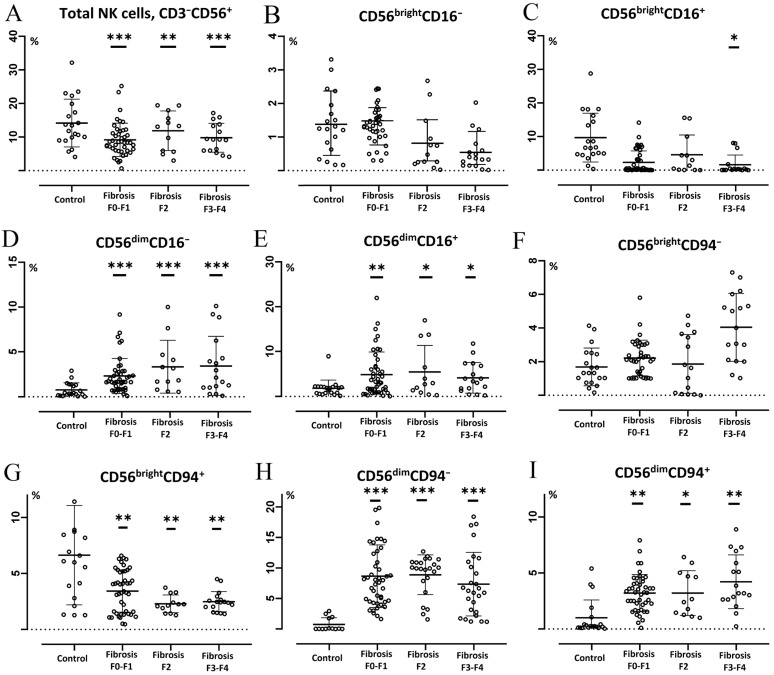
Subset composition of NK cells in patients with chronic hepatitis C (HCV genotype 1). Horizontal bars depict the group medians and quartile ranges (Me (IQR)) of the quantitative data (% NK cell subsets within total lymphocytes population). Statistical analysis was performed with the Mann–Whitney U test. Description of *p* value mark (compared to control): *—*p* value ≤ 0.05, **—*p* value ≤ 0.01, ***—*p* value ≤ 0.001. Subfigures show the distribution of NK cell subsets in groups of fibrosi of varying severity and control: (**A**)—total NK cells (CD3^−^CD56^+^), (**B**)—CD56^bright^CD16^−^, (**C**)—CD56^bright^CD16^+^, (**D**)—CD56^dim^CD16^−^, (**E**)—CD56^dim^CD16^+^, (**F**)—CD56^bright^CD94^−^, (**G**)—CD56^bright^CD94^+^, (**H**)—CD56^dim^CD94^−^, (**I**)—CD56^dim^CD94^+^.

When studying the content of various subsets of blood NK cells in CVHC patients with genotype 1, it was found that, regardless of the degree of fibrosis, patients in this group had increased relative levels of lymphocytes with the CD56^dim^CD16^−^ and CD56^dim^CD16^+^ phenotypes compared to control values (Figure 2D,E). In addition, these features were discovered against the background of a decrease in the amount of NK cells (Figure 2A). At the same time, a decrease in the proportion of CD56^bright^CD16^+^ cells relative to control values was detected only in CHCV patients with genotype 1 and degree of fibrosis F3–F4 (Figure 2C). Regardless of the degree of fibrosis, this group of patients also shows changes in the amount of NK cells expressing the CD94 marker: the content of CD56^bright^CD94^+^ cells decreases, and the levels of CD56^dim^CD94^−^ and CD56^dim^CD94^+^ cells significantly increases relative to control values (Figure 2G–I).

Regardless of the fibrosis degree, in CVHC patients with genotype 1 of HCV, the number of CD56^+^CD38^+^CD73^−^ and CD56^+^CD38^−^CD73^−^ cells in the blood decreased and the content of CD56^+^CD38^+^CD73^+^ and CD56^+^CD38^−^CD73^+^ cells increased relative to controls (Table 5). Moreover, in patients with HCV genotype 1 and METAVIR fibrosis stage F2, the amount of NK cells with the CD56^+^CD38^+^CD73^+^ phenotype was significantly reduced in comparison with the values detected in patients with METAVIR fibrosis stages F0–F1 and F3–F4.

**Figure 3:**
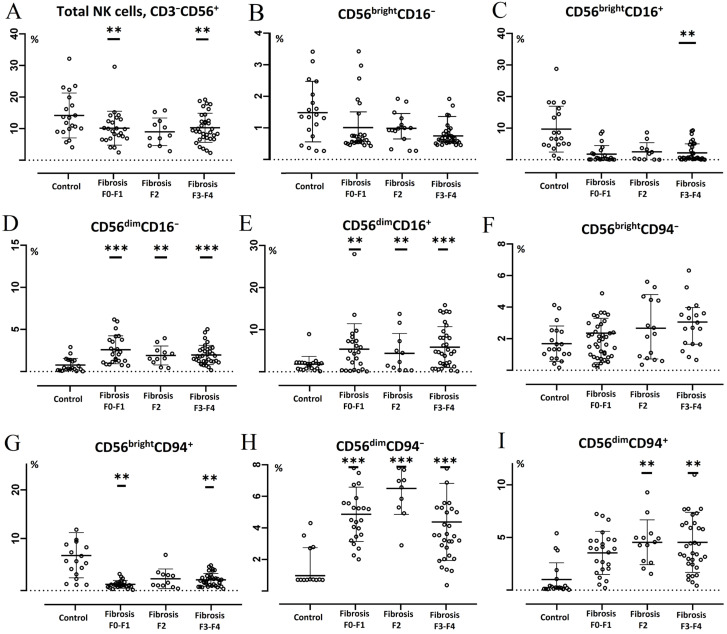
Subset composition of NK cells in patients with chronic hepatitis C (HCV genotype 3). Horizontal bars depict the group medians and quartile ranges (Me (IQR)) of the quantitative data (% NK cell subsets within total lymphocytes population). Statistical analysis was performed with the Mann–Whitney U test. Description of *p* value mark (compared to control): **—*p* value ≤ 0.01, ***—*p* value ≤ 0.001. Subfigures show the distribution of NK cell subsets in groups of fibrosi of varying severity and control: (**A**)—total NK cells (CD3^−^CD56^+^), (**B**)—CD56^bright^CD16^−^, (**C**)—CD56^bright^CD16^+^, (**D**)—CD56^dim^CD16^−^, (**E**)—CD56^dim^CD16^+^, (**F**)—CD56^bright^CD94^−^, (**G**)—CD56^bright^CD94^+^, (**H**)—CD56^dim^CD94^−^, (**I**)—CD56^dim^CD94^+^.

When studying the composition of subset and phenotype of NK cells in CVHC patients with genotype 3, depending on the severity of fibrosis, it was revealed that the number of NK cells decreased in people with METAVIR fibrosis stages F0–F1 and F3–F4 compared with control values (Figure 3A). The content of CD56^bright^CD16^+^ cells decreased in genotype 3 CVHC patients only with METAVIR fibrosis stage F3–F4 (Figure 3C). In addition, in these patients with METAVIR fibrosis stages F0–F1 and F3–F4, the level of CD56^bright^CD94^+^ cells decreases relative to control values (Figure 2G), while with METAVIR fibrosis stages F2 and F3–F4 an increase in the level of CD56^dim^CD94^+^ cells was observed (Figure 2I). Moreover, the number of NK cells with this phenotype in patients with fibrosis stage F3–F4 also significantly exceeded the level detected in individuals with fibrosis stage F0–F1 (*p* = 0.024). Regardless of the fibrosis degree, in patients with HCV genotype 3, the content of CD56^dim^CD16^−^, CD56^dim^CD16^+^ and CD56^dim^CD94^−^ cells increased relative to control values (Figure 2D,F,H). At the same time, there was a statistically significant increase in the level of CD56^dim^CD16^+^ cells in persons with METAVIR fibrosis stage F3–F4 compared to the values found in individuals with METAVIR fibrosis stage F2 (*p* = 0.018).

In patients with genotype 3 of HCV, the blood content of NK cells expressing CD38 and CD73 varied slightly among individuals with different degrees of fibrosis (Table 6). Thus, regardless of the degree of fibrosis in this group of patients, the amount of CD56^+^CD38^+^CD73^−^ and CD56^+^CD38^−^CD73^−^ cells decreased, but the level of CD56^+^CD38^+^CD73^+^ and CD56^+^CD38^−^CD73^+^ cells increased. At the same time, in patients with METAVIR fibrosis stage F3–F4, the maximum content of NK cells with the CD56^+^CD38^+^CD73^+^ phenotype was detected, which showed a statistically significant increase in comparison with the levels found in individuals with METAVIR fibrosis stage F2.

We also investigated the distribution features of the subset composition and phenotype of blood NK cells in patients with CVHC at each fibrosis stage, depending on the HCV genotype. There were no statistically significant differences in these indicators when comparing fibrosis stages F0–F1 and F3–F4. At the same time, among individuals with METAVIR fibrosis stage F2, the percentage of NK cells in the blood with the CD56^+^CD38^−^CD73^+^ phenotype in CVHC patients with genotype 3 was lower than in persons with genotype 1 of HCV (see Table 5 and Table 6).

## 4. Discussion

It is known that NK cells are heterogeneous in population, exhibit natural cytotoxic activity towards virus-infected and malignant cells, and are also capable of producing a wide range of cytokines [16,18,25]. We identified the main NK cells subsets by the expression of CD16 and CD56. CD16 is a low-affinity receptor for immunoglobulin G type III (FcγRIII) that mediates the cellular antibody-dependent cytotoxicity mechanism [26,27]. Glycoprotein CD56 (NCAM, NKH-1, Leu-19) belongs to the immunoglobulin superfamily and participates in intercellular contacts [28,29]. Based on the CD56 expression level, 2 main NK cells subsets are distinguished. CD56^bright^ NK cells characterized by the ability to actively proliferate, exhibit minimal cytotoxic activity, but can intensively synthesize and secrete cytokines such as interferon-γ, tumor necrosis factor and granulocyte-macrophage colony-stimulating factor [30]. Therefore, this subset of NK cells is defined as cytokine-synthesizing. There is also evidence that CD56^bright^ NK cells can be defined as regulatory cells (iNK) due to the pleiotropic function of cytokines in different immune and non-immune processes [31,32]. CD56^bright^CD16^+^ cells are mature NK cells, while CD56^bright^CD16^−^ cells are defined as less mature and predominantly localize in secondary lymphoid organs (due to the CCR7 expression) [25,31]. The degree of CD56^dim^ NK cell proliferation in response to activation stimuli is much lower. These cells produce a small number of cytokines (including interferon-γ) but have a high level of cytotoxicity (they are characterized as cytotoxic NK cells) [31,33]. CD56^dim^CD16^−^ cells are characterized as maturing while CD56^dim^CD16^+^ NK cells predominantly circulate in the blood, express CXCR1, CX3CR1 and ChemR23, and migrate to areas of immunoinflammatory processes [31,34].

The number of NK cells and their subset composition in patients with CVHC in our work did not differ depending on the HCV genotype, but had some features depending on the degree of liver fibrosis. Thus, the content of the total fraction of NK cells was decreased in CVHC patients with genotypes 1 and 3 of HCV: in genotype 1 persons with all fibrosis stages, and in genotype 3 individuals with liver fibrosis stage F3–F4. The level of mature cytokine-producing NK cells (CD56^bright^CD16^+^) among CVHC patients with genotypes 1 and 3 was decreased only in persons with fibrosis stage F3–F4, while the level of cytotoxic NK cells (CD56^dim^CD16^+^ and CD56^dim^CD16^−^) was increased relative to control values in all examined individuals with CVHC. The established redistribution in the subset composition of NK cells was predominantly characterized by disturbances in the ratio between cytokine-producing and cytotoxic NK cells in severe liver fibrosis and did not depend on the HCV genotype.

We also examined the amount of NK cells expressing the CD94 receptor. This receptor is a type II transmembrane glycoprotein weighing 30 kDa, and belonging to the Ca^2+^-dependent lectins family (type C) [18,35]. CD94 binds to the NKG2 family member, forming a disulfide-linked NK cell receptor for MHC class I molecules, which has higher specificity than the killer cell inhibitory/activating receptors (KIR/KAR) belonging to the Ig superfamily [35,36]. It was established that the level of synthesis of functional molecules (granzymes and perforins) depends on the CD94 receptor expression; in particular, NK cells with the CD94^+^CD56^dim^ phenotype had lower levels of expression of perforin and granzyme B and were characterized by a lower level of cellular cytotoxicity than CD94^−^CD56^dim^ cells [32]. The level of cytokine-producing NK cells with expression of the CD94 receptor in patients with HCV genotype 1 was decreased regardless of the fibrosis stage, while in patients with HCV genotype 3, a decrease in this fraction of NK cells was observed at METAVIR fibrosis stages F0–F1 and F3–F4. The number of cytotoxic NK cells that do not express the CD94 receptor was increased relative to control values in CVHC patients with genotypes 1 and 3 of HCV, regardless of the degree of fibrosis. The content of cytotoxic NK cells with CD94 expression in persons with HCV genotype 1 was also increased regardless of the degree of fibrosis, while in individuals with HCV genotype 3, the number of NK cells of this phenotype increased only at METAVIR fibrosis stages F2 and F3–F4. It should also be noted that in healthy people the level of CD56^dim^CD94^−^ NK cells was 2 times higher than the number of CD56^dim^CD94^+^ cells, and that with severe fibrosis in CVHC patients with HCV genotype 3, this ratio approached one and was minimal in persons with HCV genotype 1. Consequently, if the change in the number of cytokine-producing and cytotoxic cells, non-expressing and expressing the CD94 molecule, in CVHC, regardless of the HCV genotype, was more consistent with the mechanism of the immune response during this viral infection, then the ratio between cytotoxic NK cells with the absence and presence of CD94 receptor expression showed a greater dependence on the severity of liver fibrosis.

In patients with CVHC, the blood levels of NK cells expressing CD38 and CD73 receptors were also studied. CD38 is a glycoprotein with a molecular mass of ~45 kDa, which is expressed on the surface of many immune system cells and is defined as a glycohydrolase (EC 3.2.2.6) which catalyzes the degradation of NAD^+^ or NADP^+^ to form cyclic ADP-ribose and nicotinamide [37]. These reaction products are necessary for the intracellular Ca^2+^ pool regulation. CD38 is involved in cellular metabolism through regulation of the NAD pool and in the pathogenesis of many conditions, including diabetes, obesity, heart disease, asthma, aging and inflammation. It has been shown that expression of the CD38 receptor on the NK cell membranes leads to a decrease in their functional activity [38]. CD73 (NT5E) is the enzyme ecto-5’-nucleotidase (EC 3.1.3.5) that cleaves adenosine monophosphate to adenosine [39,40]. It has been proven that NK cells expressing CD73 have depressed cytotoxic activity and can perform regulatory cell functions [40]. In the CVHC patients we examined, the content of NK cells expressing CD38 and CD73 receptors changed significantly compared with that detected in the control group, while the identified features were weakly dependent on the HCV genotype. Firstly, in patients with CVHC, the blood content of NK cells that do not express CD38 and CD73, that is, natural killer cells with a high level of functional activity, decreased. Secondly, in the examined patients, the number of NK cells with co-expression of CD38 and CD73 increased, that is, cells with reduced functional activity that can implement a regulatory function. Moreover, the maximum number of CD38^+^CD73^+^ NK cells was detected in CVHC patients with both HCV genotypes and METAVIR fibrosis stage F3–F4. Thirdly, in patients with CVHC, again regardless of the HCV genotype, the number of major subset NK cells CD38^+^CD73^−^ decreased, the level of NK cells with the CD38^−^CD73^+^ phenotype increased in comparison with control values. These changes manifested themselves in the examined patients, regardless of the degree of fibrosis. However, in CVHC patients with HCV genotype 3 and fibrosis stage F2, the number of CD38^−^CD73^+^ NK cells was decreased compared to the level detected at this degree of fibrosis in patients with HCV genotype 1. Based on all the results obtained, this feature in the number of NK cells with the CD38^−^CD73^+^ phenotype in patients cannot be associated with a dependence on the HCV genotype. In general, we can conclude that during CVHC the number of NK cells with the expression of ecto-5’-nucleotidase increased. Moreover, the inhibitory effect of purinergic regulation on NK cells was also proven using the example of viral infections [41], and also in patients with severe fibrosis, the level of CD38^+^CD73^+^ NK cells increased.

## 5. Conclusions

At the first stage of our work, we found a significant prevalence of liver fibrosis METAVIR stages F2 and F3–F4 in CVHC patients with genotype 3 in comparison with individuals with genotype 1 of HCV. After this, we studied the phenotype and subset composition of blood NK cells for a possible explanation of the results obtained. As a result, from our point of view, we verified the concept of an increase in NK cell dysfunction parallel to the development of liver fibrosis in patients with CVHC, but the methods used at this stage focused our attention on the commonality of the immunological mechanisms of the pathological process in both groups of individuals with genotype 1 and genotype 3 of HCV.

**Figure 4:**
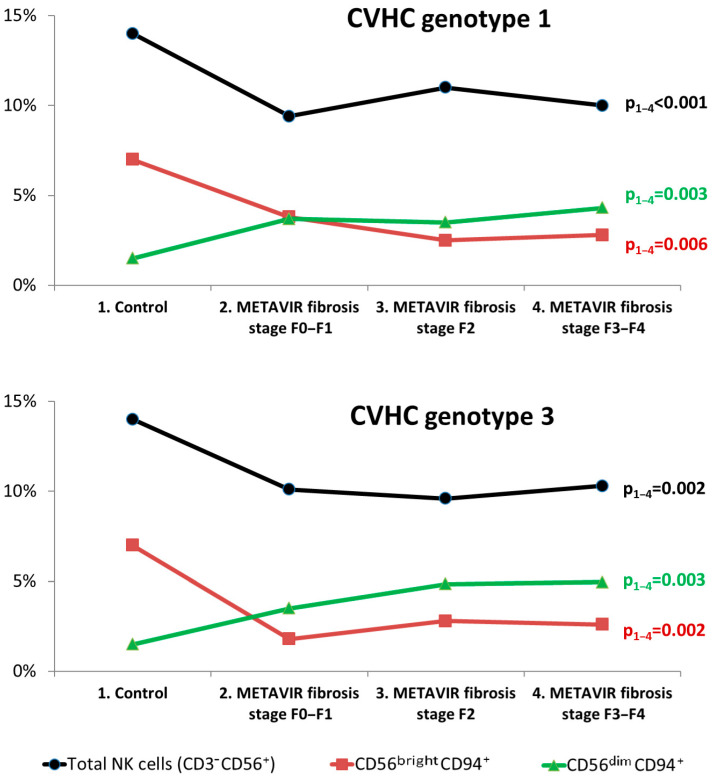
Dynamics of cytokine-producing and cytotoxic NK cells in CVHC patients with genotypes 1 and 2 depending on liver fibrosis. P_1-4_ is comparison reliability of control persons and CVHC patients with METAVIR fibrosis stage F3-F4.

Interesting features of our work include the demonstration of a violation of the relationship between cytokine-producing and cytotoxic NK cells in severe liver fibrosis in CVHC patients with both genotypes (Figure 4). Another original aspect of our study is to determine the increased level of CD94+ and CD38+CD73+ NK cells in the blood in CVHC patients with severe liver fibrosis, with both 1 and 3 genotypes of HCV, which determines the low functional activity of NK cells (due to a decrease in synthesis of perforins and granzymes). Thus, the body’s tendency to activate effector mechanisms is accompanied by a decrease in regulatory function and an increase in the action of inhibitory mechanisms that block the function of NK cells. We hope that the results obtained will be useful for planning new studies and developing methods for treating liver fibrosis and preventing the development of hepatocellular carcinoma.

## Figures and Tables

**Table 1:** Panels of monoclonal antibodies (indicating fluorochrome, protein clone and catalog number) used for cytometric analysis of NK cells.

Antibodies	Fluorochrome	Protein Clone	Catalog Number
Panel 1			
CD3	Alexa Fluor 700	UCHT1	B10823
CD16	Phycoerythrin-cyanin 7	3G8	6607118
CD45	Alexa Fluor 750	J33	A79392
CD56	R-Phycoerythrin-cyanine 5.1	N901	A07789
CD94	Phycoerythrin	R34.34	IM1980U
Panel 2			
CD3	Alexa Fluor 700	UCHT1	B10823
CD38	Fluorescein isothiocyanate	T16	A07778
CD45	Alexa Fluor 750	J33	A79392
CD56	R-Phycoerythrin-cyanine 5.1	N901	A07789
CD73	Phycoerythrin	AD-2	B68176

All monoclonal antibodies were produced by Beckman Coulter (Indianapolis, IN, USA).

**Table 2:** Anthropometric, anamnestic and laboratory parameters of CVHC patients with genotypes 1 and 3 of HCV.

Parameters	CVHC with Genotype 1 Total (*n* = 297)	CVHC with Genotype 3 Total (*n* = 231)	*p*
Men/women, n	164/133	125/106	0.800
Age (years), Me (IQR)	41 (38−46)	41 (38−45)	0.623
BMI, kg/m^2^ Me (IQR)	24 (22−25)	25 (22−27)	0.518
Tobacco smoking, n (%)	17 (5.7%)	14 (6.1%)	0.871
Alcohol consumption, n (%)	33 (11.1%)	24 (10.4%)	0.791
White blood cells, 10^9^/L Me (IQR)	6.40 (5.26−8.33)	6.58 (5.40−8.36)	0.234
Neutrophils, % Me (IQR)	54.4 (48.8−60.0)	55.4 (48.8−60.0)	0.932
Lymphocytes, % Me (IQR)	33.3 (28.7−39.1)	33.3 (29.7−38.7)	0.702
Platelets, 10^9^/L Me (IQR)	230 (202−280)	239 (201−283)	0.906
ALT, IU Me (IQR)	68 (30−85)	70 (39.2−142)	0.033
Albumin, g/L Me (IQR)	42 (38−45)	41 (38−44)	0.212
Bilirubin, µmol/L Me (IQR)	15.4 (11.5−25.6)	15.0 (12.0−23.4)	0.504

Quantitative indicators are presented as median and interquartile ranges (Me (IQR)). Qualitative data are presented in absolute values and percentages (n (%)). The significance of the differences was calculated using the Mann–Whitney U test (for quantitative indicators) and the Fisher’s exact test (for qualitative variables). BMI is body mass index; ALT is alanine aminotransferase; IU is international unit.

**Table 3:** Anthropometric, anamnestic characteristics and laboratory parameters of CVHC patients with genotypes 1 and 3 of HCV which determined the NK cell phenotype.

Parameters	CVHC with Genotype 1 Total (*n* = 74)	CVHC with Genotype 3 Total (*n* = 69)	Control Group (*n* = 20)
Age (years), Me (IQR)	43 (38−47)	42 (38−46)	42 (37−47)
		p_1–2_ = 0.849	p_1–3_ = 0.868 p_2–3_ = 0.934
BMI, kg/m^2^ Me (IQR)	24 (22−26)	25 (22−27) p_1–2_ = 0.831	24 (21−27) p_1–3_ = 0.883 p_2–3_ = 0.911
Tobacco smoking, n (%)	4 (5.4%)	4 (5.8%) p_1–2_ = 0.919	1 (5.0%) p_1–3_ > 0.943 p_2–3_ = 0.892
Alcohol consumption, n (%)	8 (10.8%)	7 (10.1%) p_1–2_ = 0.897	0 (0.0%) p_1–3_ = 0.125 p_2–3_ = 0.138
White blood cells, 10^9^/L Me (IQR)	6.42 (5.14−8.32)	6.60 (5.31−8.39) p_1–2_ = 0.623	6.23 (4.98−7.56) p_1–3_ = 0.541 p_2–3_ = 0.460
Neutrophils, % Me (IQR)	54.7 (48.2−60.5)	55.2 (49.1−59.8) p_1–2_ = 0.973	57.4 (50.9−63.1) p_1–3_ = 0.243 p_2–3_ = 0.319
Lymphocytes, % Me (IQR)	33.5 (28.5−39.4)	33.2 (29.3−39.1) p_1–2_ = 0.962	29.8 (23.7−34.5) p_1–3_ = 0.168 p_2–3_ = 0.194
Platelets, 10^9^/L Me (IQR)	232 (202−282)	238 (200−284) p_1–2_ = 0.926	247 (205−297) p_1–3_ = 0.788 p_2–3_ = 0.823
ALT, IU Me (IQR)	67 (29−86)	70 (38−145) p_1–2_ = 0.217	22 (13−30) p_1–3_ = 0.032 p_2–3_ = 0.008
Albumin, g/L Me (IQR)	42 (38−46)	40 (37−44) p_1–2_ = 0.576	44 (39−48) p_1–3_ = 0.361 p_2–3_ = 0.122
Bilirubin, µmol/L Me (IQR)	15.3 (11.5−25.2)	15.1 (11.8−23.9) p_1–2_ = 0.681	10.8 (5.3−15.6) p_1–3_ = 0.031 p_2–3_ = 0.038

Quantitative indicators are presented as median and interquartile ranges (Me (IQR)). Qualitative data are presented in absolute values and percentages (n (%)). The significance of the differences was calculated using the Mann–Whitney U test (for quantitative indicators) and the Fisher’s exact test (for qualitative variables); p_1–2_ and p_1–3_—significant difference compared with the indicators of CVHC patients with HCV genotype 1; p_2–3_—significant difference compared with the indicators of CVHC patients with HCV genotype 3. BMI is body mass index; ALT is alanine aminotransferase; IU is international unit.

**Table 4:** Frequency of various stages of liver fibrosis in CVHC patients with genotypes 1 and 3 of HCV (n (%)).

Liver Fibrosis	METAVIR Fibrosis Stage F0–F1	METAVIR Fibrosis Stage F2	METAVIR Fibrosis Stage F3–F4
CVHC patients with HCV genotype 1 (*n* = 297)	201 (67.7%)	35 (11.8%)	61 (20.5%)
CVHC patients with HCV genotype 3 (*n* = 231)	107 (46.3%)	49 (21.2%)	75 (32.5%)
OR (CI)	0.41 (0.29–0.59) *p* < 0.001	2.02 (1.26–3.23) *p* = 0.004	1.86 (1.25–2.76) *p* = 0.003

Significance in indicators was calculated using the odds ratio (OR) and confidence interval (CI).

**Table 5:** The content of NK cells expressing CD38 and CD73 in the blood (%) in patients with chronic hepatitis C (HCV genotype 1) (Me (IQR)).

Parameters	Control	Patients with CVHC		
		Fibrosis F0–F1	Fibrosis F2	Fibrosis F3–F4
CD56 ^+^ CD38^−^CD73^−^	1.46 (1.19–3.01)	0.75 (0.26–1.23) p_1_ < 0.001	0.84 (0.52–1.00) p_1_ = 0.016	0.77 (0.55–1.34) p_1_ = 0.021
CD56^+^CD38^−^CD73^+^	0.001 (0.0–0.011)	0.065 (0.030–0.125) p_1_ < 0.001	0.121 (0.064–0.144) p_1_ < 0.001	0.059 (0.051–0.121) p_1_ < 0.001
CD56^+^CD38^+^CD73^−^	10.56 (5.51–14.82)	5.95 (3.14–9.49) p_1_ = 0.020	4.63 (1.42–6.96) p_1_ = 0.011	3.45 (1.82–10.64) p_1_ = 0.023
CD56^+^CD38^+^CD73^+^	0.001 (0.0–0.102)	0.631 (0.191–0.845) p_1_ < 0.001	0.234 (0.075–0.330) p_1_ < 0.001 p_2_ = 0.024	0.809 (0.386–1.300) p_1_ < 0.001 p_3_ = 0.007

Comments: The quantitative indicators (NK cell subsets % within total lymphocytes population) are presented as median and quartile ranges (Me (IQR)); p_1_—statistically significant differences versus controls; p_2_—statistically significant differences versus patients with Fibrosis F0–F1; p_3_—statistically significant differences versus patients with Fibrosis F2.

**Table 6:** The content of blood NK cells expressing CD38 and CD73 (%) in patients with CVHC (HCV genotype 3) (Me (IQR)).

Parameters	Control	Patients with CVHC		
		Fibrosis F0–F1	Fibrosis F2	Fibrosis F3–F4
CD56^+^CD38^−^CD73^−^	1.46 (1.19–3.01)	0.82 (0.26–1.36) p_1_ = 0.008	0.63 (0.28–1.21) p_1_ = 0.004	0.62 (0.37–1.39) p_1_ = 0.002
CD56^+^CD38^−^CD73^+^	0.001 (0.0–0.011)	0.053 (0.044–0.122) p_1_ < 0.001	0.045 (0.042–0.057) p_1_ < 0.001	0.068 (0.028–0.104) p_1_ < 0.001
CD56^+^CD38^+^CD73^−^	10.56 (5.51–14.82)	5.13 (3.39–6.45) p_1_ = 0.004	1.51 (0.94–5.84) p_1_ = 0.004	5.62 (2.58–7.63) p_1_ = 0.004
CD56^+^CD38^+^CD73^+^	0.001 (0.0–0.102)	0.498 (0.128–0.768) p_1_ < 0.001	0.310 (0.152–0.532) p_1_ < 0.001	0.674 (0.421–1.163) p_1_ < 0.001 p_3_ = 0.004

Comments: The quantitative indicators (NK cell subsets % within total lymphocytes population) are presented as median and quartile ranges (Me (IQR)); p_1_—statistically significant differences versus controls; p_3_—statistically significant differences versus patients with Fibrosis F2.

## Data Availability

Availability of data and materials. If you need clarifications, or need additional information, you can write to the email: gastro@impn.ru.
